# The impact of the Family Medicine Model on patient satisfaction in Turkey: Panel analysis with province fixed effects

**DOI:** 10.1371/journal.pone.0210563

**Published:** 2019-01-30

**Authors:** Susan P. Sparkes, Rifat Atun, Till Bӓrnighausen

**Affiliations:** 1 Department of Global Health and Population, Harvard T.H. Chan School of Public Health, Boston, MA, United States of America; 2 Heidelberg Institute of Global Health, Faculty of Medicine and University Hospital, University of Heidelberg, Heidelberg, Germany; University of Botswana Faculty of Medicine, BOTSWANA

## Abstract

**Background:**

In this study, we aim to establish the impact of the introduction of the Family Medicine Model patient satisfaction in the Turkish health system.

**Methods:**

We use data on data 69,028 primary health care (PHC) patients over the period 2010–2012. We estimate the impact of the Family Medicine Model in panel regressions with province fixed effects, exploiting the sequential introduction of this health systems transformation across Turkey's 81 provinces. We use principal component analysis to reduce the dimensionality of the data from the European Patients Evaluate General/Family Practice (EUROPEP) patient satisfaction survey, to focus on the fundamental dimensions of patient satisfaction and to decrease the need for multiple hypothesis testing. We identified two key principal components. The first captured primarily information on satisfaction with provider behavior and the second on satisfaction with the organization of care. We then use these two principal components as outcome variables in our panel analysis to estimate the causal impact of the introduction of the Family Medicine Model.

**Results:**

The Family Medicine Model significantly improved patient satisfaction across a range of dimensions. The coefficient results showed a positive and statistically significant impact (p-values<0.05) of the Family Medicine Model on the outcome variables representing the satisfaction dimensions clinical behaviour and the organization of care even after controlling for calendar time fixed effects.

**Conclusions:**

The introduction of the Family Medicine Model in Turkey, which was primarily aimed at achieving universal health coverage goals, substantially improved patient satisfaction. This study provides some of the first national-level evidence that the introduction of a Family Medicine Model can substantially improve patient satisfaction.

## Introduction

Patient satisfaction is central to assessing the performance of primary health care (PHC) and how that care responds to needs, where preventive services and most care for chronic illness is delivered [1]. While population health outcomes and financial risk protection are objective measures of health system performance, patient satisfaction relies on subjective assessments of the health system by patients, reflecting the perceived fulfillment of their needs and desires through the use of healthcare services [2–4]. Improvement in patient satisfaction is important because of its intrinsic value, its instrumental function in improving retention and adherence amongst patients, and also its indication of public support for health policy and policy makers [5–9]. Yet, there are no studies that have established the causal impacts of health systems changes and transformation on patient satisfaction. Rather, research on patient satisfaction has correlated different types of health systems or socio-demographic characteristics with varying levels of patient satisfaction [2, 10–17].

In this study, we estimate for the first time the causal impact of a major health system transformation–the so-called Family Medicine Model, a family medicine-centred PHC transformation of the Turkish health system–on patient satisfaction. To this end, we use province fixed effects panel analysis applied to provincially-representative panel data on a range of patient satisfaction outcomes in Turkey over the period 2010–2012. The introduction of the Family Medicine Model was part of Turkey’s broader Health Transformation Programme (2003–2012) aimed at achieving universal health coverage [18]. Unlike the other elements of this national health transformation, the Family Medicine Model was introduced sequentially across the 81 provinces in Turkey between 2005 and 2011 [18].

One of the primary motivations behind the Family Medicine Model was to improve patient satisfaction [18–20]. PHC in Turkey was underfunded relative to secondary and tertiary care, and consequently facilities lacked adequate human and operational resources, as well as necessary equipment and supplies [21, 22]. As a result, PHC was characterised by long waiting times, poor quality and low levels of utilization [23].

Turkey’s Family Medicine Model consisted of five specific policy interventions. First, the Ministry of Health dedicated additional financial resources to PHC. Between 2002 and 2007 alone, expenditures on PHC and preventive services tripled [23]. Second, the Ministry of Health created a new designation for general practitioners by requiring an additional ten-day training program in family medicine. Upon completion of this training, these physicians were categorized as family medicine specialists. Third, these newly trained family medicine specialists were contracted for two-years using a mixed provider payment system. The new payment system combined per capita remuneration with performance-related pay, which rewarded good performance regarding 3 maternal and child health indicators and 35 service delivery indicators [22]. Under this mixed payment system, remuneration of family medicine specialists could be reduced by 20% if they failed to meet performance targets and their contracts could be terminated if they did not keep their enrolled patient list above 1,000 [22, 24]. Fourth, every Turkish citizen was enrolled as a patient of a named family medicine specialist. Patients could exercise choice to select their family medicine specialist. As a result, family medicine specialists faced incentives to both attract patients and ensure that patients on their lists did not switch to a different family physician. Fifth, the Ministry of Health increased the number of PHC facilities, and transformed them into family medicine centers, to expand coverage in underserved areas of the country, namely rural areas and south east Turkey [25].

As it was initially envisioned by policymakers, family medicine specialists were supposed to act as gatekeepers to the secondary and tertiary care services. However, the gatekeeping component of the reform was abandoned due to political opposition [19] and individuals could continue to bypass PHC services to seek care directly at hospitals and specialty clinics

While other studies have compared average patient satisfaction ratings with PHC services in Turkey between 2010 and 2012, as well as with the broader health systems transformation [26–29], the analysis presented in this paper shows for the first time how these average levels changed as a result of a specific intervention–the Family Medicine Model. For this purpose, we use a quasi-experimental approach to causal impact estimation–panel analysis with province fixed effects–which allows us to control results for all time-invariant province-level confounders of the relationship between the exposure (the Family Medicine Model) and satisfaction outcomes.

## Methods

### Intervention exposure

**Fig 1:**
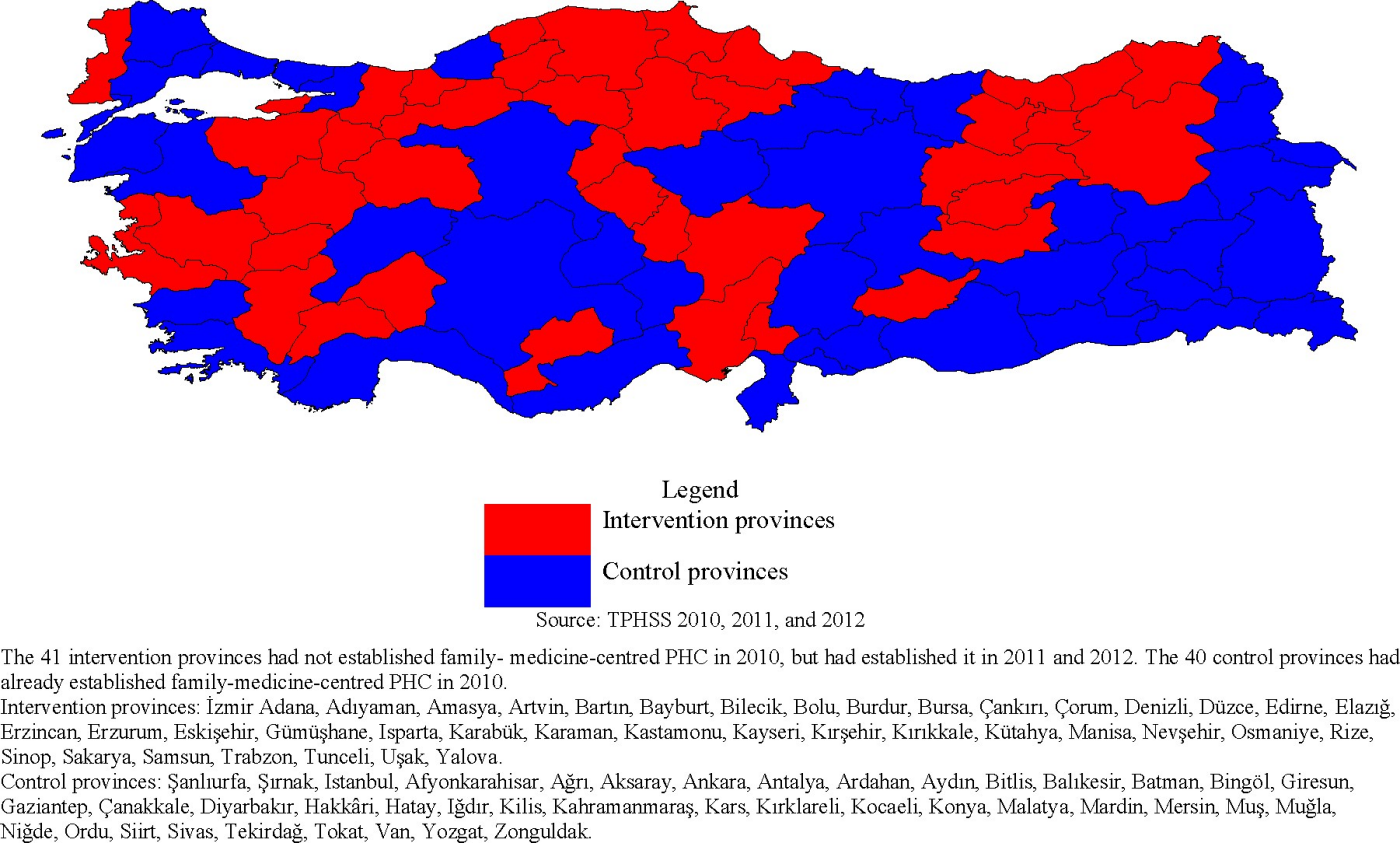
Intervention and control provinces.

As of December 2009 (EUROPEP-Turkey Patient Satisfaction with Health Services Survey (EUROPEP-TPSHSS) 2010), 40 of Turkey’s 81 provinces had fully implemented the Family Medicine Model and the remaining 41 provinces introduced it after December 2009 (Fig 1). By December 2010 (EUROPEP-TPSHSS 2011 and 2012) all 81 provinces in Turkey had fully implemented the Family Medicine Model (Fig 1) [30]. The period of time between introduction and full implementation is six months, as determined by the Turkish Ministry of Health. Of the 41 provinces that introduced the Family Medicine Model after December 2009, eight provinces began introducing it between December 2009 and June 2010, when the baseline survey data was collected (five provinces in January 2010, two in April 2010, and one in May 2010) [31].

### Outcomes data and variables

The outcomes data for this analysis were derived from three provincially-representative EUROPEP-TPSHSS. The Ministry of Health of the Republic of Turkey, General Directorate for Health Data oversaw data collection efforts and received related ethics approval (Project Reference no. A.4.4.1/ SAGEM/17). Oral consents of the patients willing to participate in the study were taken. These surveys used the EUROPEP Patient Satisfaction Scale, which consists of 23 standard and internationally comparable questions asking patients to make assessments of general practice care (see Table 1 for individual questions) [12, 32–34]. The sampling frame for the EUROPEP-TPSHSS included family practice centres in all 81 Turkish provinces in each of the three years 2010, 2011 and 2012. The list of these facilities was provided by provincial health directorates. The EUROPEP-TPSHSS used a multi-step random sampling approach to ensure representativeness at both the national and provincial levels. In the first step, 1,501 primary health care facilities (including both health centres and Family Medicine Units) were randomly selected from a list of local health directorates. Next, primary care facility-days were randomly selected among the selected facilities and every second patient visiting the practices on the selected days was interviewed [27]. An interview-based approach was used so as to ensure no bias was introduced due to literacy issues amongst lower socio-economic groups using the facilities. To reduce bias introduced by this in-person approach, each province trained expert pollsters to administer the survey questionnaire [33]. The data forms a provincially-representative panel data set [35]. Oral consents of the patients willing to participate in the study were taken.

**Table 1:** Summary statistics for independent variables.

Variable	2010				2011				2012	
	Control Provinces*		Intervention Provinces*		All Provinces		All Provinces		All Provinces	
	Frequency (N)	Relative Frequency (%)	Frequency (N)	Relative Frequency (%)	Frequency (N)	Relative Frequency (%)	Frequency (N)	Relative Frequency (%)	Frequency (N)	Relative Frequency (%)
**Sex**										
**Male**	4,498	50.6	4,944	48.7	9,442	49.6	11,196	50.2	13,873	49.9
**Female**	4,388	49.48	5,210	51.1	9,598	50.4	11,090	49.8	13,906	50.1
**Education**										
**Illiterate**	713	8.0	1,070	10.5	1,783	9.4	1,938	8.7	2,499	9.0
**Primary education**	4,389	49.4	4,755	46.8	9,144	48.0	12,542	56.3	15,090	54.3
**High school education**	2,445	27.5	2,953	29.1	5,398	28.4	5,036	22.6	6,141	22.1
**Bachelor/post-graduate education**	1,339	15.1	1,376	13.6	2,715	14.3	2,770	12.4	4,049	14.6
Place of residence**										
**Urban**	6,251	69.9	6,135	60.4	12,350	64.9	20,828	93.5	24,807	89.3
**Rural**	2,671	30.1	4,019	39.6	6,690	35.1	1,458	6.5	2,972	10.7
**Family medicine implemented**										
**No**					10.154	53.3	0	0	0	0
**Yes**					8,886	46.7	22,286	100	27,779	100
	Mean	Standard Deviation	Mean	Standard Deviation	Mean	Standard Deviation	Mean	Standard Deviation	Mean	Standard Deviation
**Age**	41.0	15.3	37.8	14.1	39.3	14.7	39.3	15.1	40.4	15.5
Socioeconomic Development Index***	12.3	6.1	16.7	7.5						

*’Control’ represents all provinces that had fully implemented the Family Medicine Model in 2010 and ‘Intervention’ represents all provinces that had not yet fully implemented the Family Medicine Model in 2010.

**The classification for urban and rural was altered between 2010 and 2011/2012. In 2010, four categories were used to classify whether an individual resided in an urban or rural area. There is a difference in frequencies in 2010 as compared to 2011/2012 as a result of this discrepancy in classification. Sampling was consistent within years across the urban/rural categories and therefore we include it as a control variable.

***The socioeconomic development index uses ingredients analysis to develop a composite index by bringing together population-based representative survey data from 2009 and 2010 on 61 parameters grouped into eight categories, namely: demographic (five parameters); education (six); health (five); employment (eight); competitiveness and innovation capacity (15); fiscal capacity (seven); access (six); and life satisfaction (nine). Data are from The Republic of Turkey, Ministry of Development, Directorate General of Regional Development and Structural Adjustment; Monitoring, Evaluation, and Analysis Department, Level 2 zones, socioeconomic development ranking, May 1, 2013.

The outcome data for our analysis comes from the answers to the questions in the EUROPEP-TPSHSS. The responses for satisfaction outcomes were categorical and were coded on a five-point Likert scale: “very bad/very dissatisfied” (1), “bad/dissatisfied” (2), “neither/neutral” (3), “good/satisfied” (4), and “very good/very satisfied” (5). We used principal component analysis to reduce the dimensionality of the EUROPEP patient satisfaction survey data [36]–to focus the analysis on a few fundamental dimensions of patient satisfaction and to decrease the need for multiple hypothesis testing using all of the 23 patient satisfaction outcomes contained in the dataset (see S1 Appendix) [6, 7, 37–41]. We used an orthogonal (varimax) rotation in the principal component analysis. To decide how many principal components to extract, we used the Kaiser criterion selecting principal components with eigenvalues of 1.0 or higher [42].

### Analysis

The staggered introduction of the Family Medicine Model across Turkish provinces over time (Fig 1) allows us to employ province fixed effects in our panel analysis of the Family Medicine Model on patient satisfaction in Turkey [43–45]. The province fixed effects control for all–observed and unobserved–time-invariant province-level confounders of the relationship between the exposure (the Family Medicine Model) and patient satisfaction. Such province-level confounders include stabile cultural preferences, the influences of location and geography, and long-term infrastructure.

All models were estimated using the statistical software package, Stata 13.0 [46]. With principal components as outcome variables, we used ordinary least squares (OLS) models in the fixed effects estimation (STATA command reg). We also used an ordered-logit model (STATA command ologit) to estimate the results for the 23 individual estimations (analysis 1) [47].

### Control variables

The province fixed effects in our analysis controlled for confounding on both all observed and all unobserved time-invariant province-level characteristics, such as geographical location, infrastructure, and local culture [48]. Additionally, we controlled for a range of potential time-varying observed confounders at the individual level–respondent’s age, sex, education, and place of residence (rural versus urban)–which have all been found to be important determinants of patient satisfaction previous studies [7, 16]. Finally, we controlled for secular trends in patient satisfaction through calendar time fixed effects. All error terms were clustered at the facility level.

### Samples and sensitivity analyses

Inclusion criteria for the EUROPEP-TPSHSS were patients visiting family practice centres, aged 18 or above, and able to answer all questions. The sample size was calculated using the square root sampling methods based on city population, urban/rural population, and male/female population ratios. Importantly, effective sample sizes were always greater than the minimum calculated for representativeness at the provincial-level in Turkey [27].

Of the total 101,903 EUROPEP-TPSHSS survey respondents (34,472 respondents in 2010, 34,764 respondents in 2011, and 32,667 respondents in 2012), 32,875 did not answer one or more survey questions. We first conducted our analyses using the sample of respondents who answered all 23 patient satisfaction questions in the EUROPEP-TPSHSS (69,028). This sample had the advantage of remaining the same when we used different satisfaction questions as outcome variables, ensuring that differences across the fixed effects estimations were entirely due to the different outcomes and not due to different samples. We used this sample to carry out the principal component analysis, and the fixed effect estimation using the principal components representing the data. We also used each of the 23 individual survey questions as outcome variables in separate fixed effects analysis. We conducted the additional analyses to test the robustness of our findings by using different sample sizes, imputing missing values, and reassigning the eight provinces that had partially introduced the Family Medicine Model between January 2010 and May 2010 to the set of control provinces. All three analyses confirmed our results, with both effect sizes and determinations of significance essentially unchanged (see S2 Appendix).

## Results

Table 1 shows summary statistics of the independent variables for the survey population in 2010, 2011 and 2012 respectively. At baseline, average patient ratings in intervention provinces were between “neutral” (3) and “satisfied” (4) for eight indicators and between “satisfied” (4) and “very satisfied” (5) for 15 indicators. Between 2010 and 2012, an increasing proportion of respondents indicated high satisfaction ratings (“satisfied” or “very satisfied”) across all survey questions and for the estimated principal components.

### Principal component analysis

The first two components identified in the principal component analysis (with Eigen value >1.0) explained 65% of the total variance in the patient satisfaction data (principal component 1 (PC1) explains 49% and principal component 2 (PC2) explains 16%). Variables that loaded heavily (≥15%) on PC1 captured different aspects of satisfaction with healthcare workers’ clinical behaviour, such as “listening to you” and “physical examination” (survey questions 1 through 17). Variables that loaded heavily (≥15%) on PC2 captured satisfaction with the processes and policies affected by the organization of care in PHC facilities operates, such as “getting an appointment to suit you” and “waiting times” (survey questions 17 through 23) (see Table a in S1 Appendix for factor loading results). These two dimensions of patient satisfaction are the same as those identified by the researchers who developed and applied the EUROPEP survey in 16 European countries, and in line with this earlier study we interpret PC1 as a measure of patient satisfaction with clinical behaviour and PC2 as a measure of patient satisfaction with organization of care [32, 36]

### Fixed effects estimations: Principal components of patient satisfaction

Table 2 presents the results of the province fixed effects panel estimations models using PC1 (satisfaction with clinical behaviour) and PC2 (satisfaction with organization of care) as outcome variables. The results that included the calendar time fixed effects were the more conservative estimates because they controlled for any variability related to time. The coefficient results showed a positive and statistically significant impact (p-values<0.05) of the Family Medicine Model on the outcome variables even after controlling for calendar time fixed effects. We found that the Family Medicine Model led to a significant increase in patient satisfaction with both clinical behaviour (PC1) and the organization of care (PC2).

**Table 2:** Results from province fixed effects panel regressions. Patient satisfaction principal components.

Variable	Patient Satisfaction Principal Component #1				Patient Satisfaction Principal Component #2			
	Clinical Behaviour	(95% CI)	Clinical Behaviour	(95% CI)	Organization of Care	(95% CI)	Organization of Care	(95% CI)
Intervention	1.90*	(1.37–2.37)	1.10*	(0.45–1.75)	1.18*	(0.83–0.52)	0·84*	(0.41–1.27)
*Year*^†^								
2010			Ref.	Ref.	-		Ref.	Ref.
2011	-		0.45	(0.29–0.70)	-		0.06	(-0.24–0.37)
2012	-		1.04*	(0.5–1.45)	-		0.55*	(0.28–0.81)
*Sex*								
Male	Ref.	Ref.	Ref.	Ref.	Ref.	Ref.	Ref.	Ref.
Female	0.12*	(0.06–0.19)	0.12*	(0.05–0.18)	0.02	(-0.02–0.05)	0·01	(-0.02–0.05)
Age	0.01*	(0.01–0.16)	0.01*	(0.01–0.02)	0.01*	(0.01–0.01)	0·01*	(0.01–0.01)
*Place of residence*								
Urban	Ref.	Ref.	Ref.	Ref.	Ref.	Ref.	Ref.	Ref.
Rural	0.00	(-0.24–0.24)	0.06	(-0.17–0.29)	0.22*	(0.06–0.37)	0·24*	(0.09–0.39)
*Education*								
Illiterate	Ref.	Ref.	Ref.	Ref.	Ref.	Ref.	Ref.	Ref.
Primary education	0.13	(-0.00–0.26)	0.10*	(-0.02–0.22)	0.09*	(0.01–0.17)	0·08	(-0.00–0.16)
High school education	-0.15*	(-0.30–0.01)	-0.16*	(-0.30–0.01)	-0.05	(-0.13–0.04)	-0·05	(-0.15–0.04)
Bachelor/post- graduate education	-0.15	(-0.34–0.02)	-0.18*	(-0.36–0.01)	-0.02	(-0.13–0.10)	-0·04	(-0.15–0.08)
Province fixed effect	Y		Y		Y		Y	
Observations	69,028		69,028		69,028		69,028	

* p<0.05

^*†*^ We cannot determine if calendar time fixed effects control only for an underlying secular trend or whether it also controls for some of the policy impact because the Family Medicine Model was rolled out in two major steps. Due to the time needed for full implementation, some of the variability controlled for through the inclusion of calendar time fixed effects may actually be attributable to the introduction of the Family Medicine Model. Therefore, we consider our point estimates reported in Table 2 to be lower and upper bounds of the impact of the introduction of the Family Medicine Model on patient satisfaction ratings. Standard errors are clustered at the province level.

### Fixed effects estimations: Satisfaction questions

**Fig 2:**
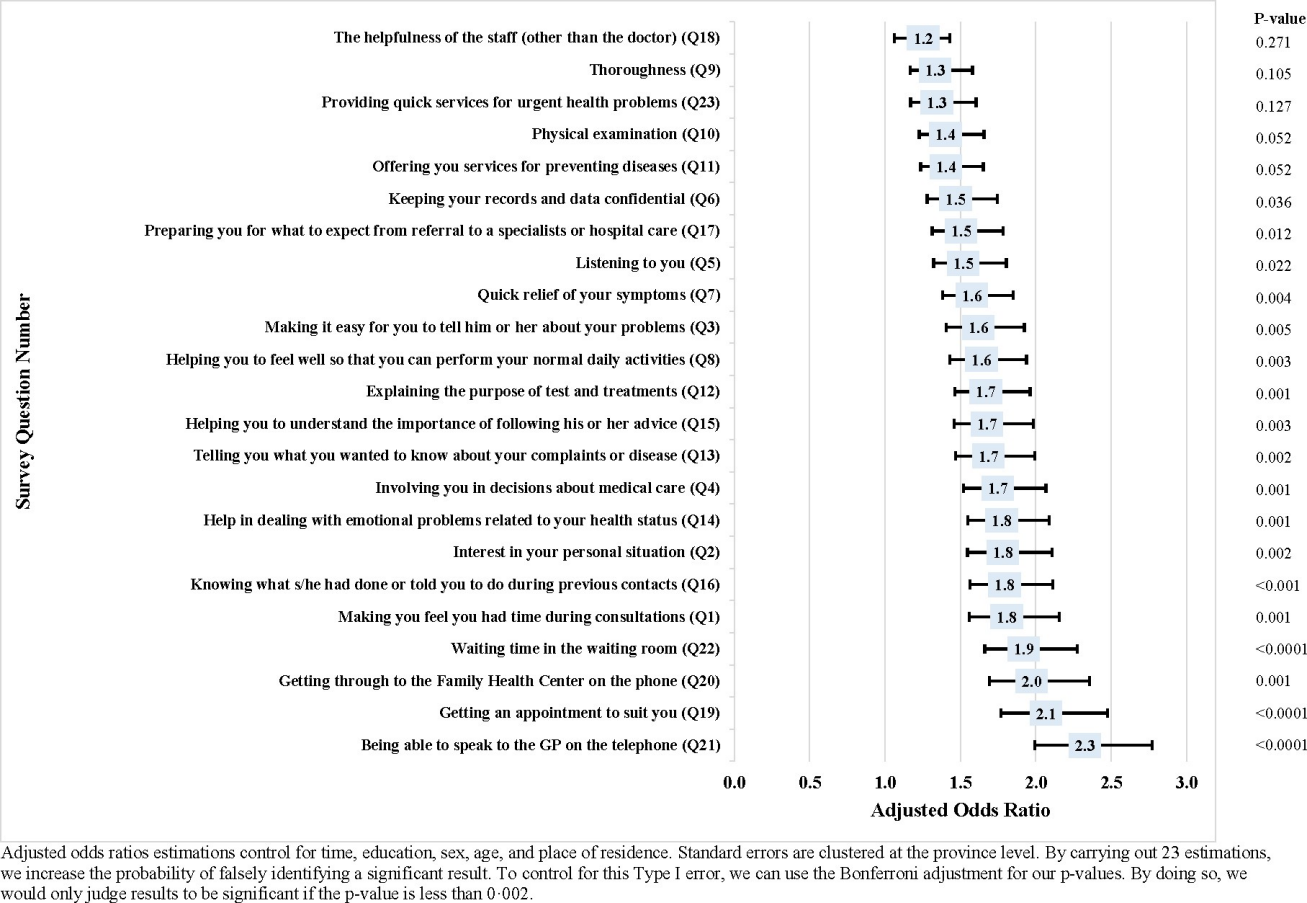
Results from province fixed effects panel regressions. Individual patient satisfaction questions.

Fig 2 shows the results of the province fixed effects panel estimations using the 23 individual patient satisfaction questions as outcome variables. The Family Medicine Model had a positive impact on patient satisfaction (Fig 2). The adjusted odds ratios for the outcome variables with statistically significant (18 of 23 outcome variables) results (p-values<0·05) ranged from 1·46 to 2.33, i.e. the introduction of the Family Medicine Model increased patient satisfaction ratings on average by about one and a half to two categories on the Likert scale of satisfaction categories for 18 of the 23 EUROPEP question outcomes. When using a stricter Bonferroni-adjusted threshold for significance due to multiple hypothesis testing (p-value<0.002), 11 of the 23 outcome variables have statistically significant results with coefficients ranging from 1.37 to 2.33 [49].

## Discussion

Our results show that a major transformation of a health system–the introduction of a Family Medicine Model in Turkey–substantially improved patient satisfaction across a wide range of dimensions. These findings are the first causally strong impact size estimates of the impact of a major national health system transformation on patient satisfaction.

Our findings are consistent with those of other studies that have found high average levels of reported satisfaction with health services in Turkey between 2010 and 2012. Using the EUROPEP-TPSHSS, Aktürk, Ateşoğlu, and Çift (2015) find that on average 88.3% of users of PHC facilities were satisfied with the services they received [27]. Bulut and Oguzoncul (2014) use the same data to examine differences in satisfaction rates with the Family Medicine Model between socioeconomic groups in a single province. They found that on average males, those who had completed secondary school education, farmers, and patients with incomes between 1,001–2,500 Turkish Lira reported significantly higher rates of satisfaction as compared to other respondents [28]. Through a cross-sectional study conducted between October 2011 and January 2012, Jadoo et al (2014) observed that more than two-thirds of respondents believed that the Health Transformation Programme had a positive impact on Turkey’s health system [29]. While these studies provide information on average satisfaction levels, they differ from our study in two critical ways. First, they do not causally examine the impact of the Family Medicine Model on satisfaction levels. Second, they treat individual satisfaction-related questions individually, and do not conduct principal component analysis to provide summary indicators to measure satisfaction.

Our quasi-experimental approach demonstrates the power of province fixed effects analysis in answering important health system questions. The combination of the step-wise rollout of the Family Medicine Model with province panel data generated from patient exit surveys allowed us to estimate causal impacts of the intervention on patient satisfaction. This approach to causal inference is a powerful analytical strategy whenever national health systems reforms are introduced sequentially over time across different sub-national communities (e.g. states, provinces or districts).

In addition to contribution to evidence, our results are particularly important for Turkey’s policymakers and citizens. Our findings demonstrate that the Ministry of Health was successful in achieving one of the primary objectives of the Family Medicine Model–improved patient satisfaction and quality perceptions–and as a result translated aspirations of “people-centred health systems” into practice. This successful implementation contributed to continued political support for the political party spearheading the transformation effort [20]. Additional studies have pointed to some weak points in terms of implementation, including coordination or care and ability to fully enroll all Turkish citizens with a family medicine practitioner, which points to the need for continued investment in ensuring the system meets patient needs [50].

Another way to use the results of this study is to inform policymakers on how to change healthcare seeking behaviour of patients. Access can be viewed as the “degree of fit” between patient expectations and the health system or “responsiveness” of a health system [4, 41]. Patient satisfaction captures important dimensions of this fit or responsiveness, and is a key link between access and actual utilization of care. To even better understand how this fit can be maintained, it will be useful to study the mechanisms linking the Family Medicine Model with satisfaction.

In this study, we evaluate the Family Medicine Model as a whole and thus cannot infer which particular component of the changes were responsible for increased patient satisfaction. Future research, including qualitative interviews with patients and health workers and participant observation, may elucidate further the mechanisms transmitting the impact of the Family Medicine Model on patient satisfaction.

### Limitations

There are important limitations to our analysis. Unobserved patient confounding factors could have biased our causal impact estimates. For instance, we cannot control perfectly control for patient health care need or those patients that chose not to seek care in PHC settings in the analysis. However, major changes in treatment need over a three-year period and on a national scale are unlikely, and, to a large extent, need will have been constant. An additional limitation is that the EUROPEP-TPSHSS questions are not anchored to a common reference point and may thus not be completely comparable across individuals and time. We control for a wide range of individual-level confounders, which have been shown to systematically affect patient satisfaction scores across a given population [38, 51]. The time period to analyse the implementation impact was also short given data availability, which limits the ability to assess the sustained impact of the reform on patient satisfaction. Therefore, the results relate to the immediate implementation impact and not how it changed over time. There is no data available for patients that refused to respond to the survey, which could introduce bias in the case that they were patients that tended to be more dissatisfied with the services they received. However, there is not a clear indication that this would be systematic bias given these patients may also want to complain if they had a strongly negative experience. As noted above, this paper does not assess the actual package of services or reforms that were introduced as part of the Family Medicine Model. Rather, it examines the totality of these interventions in the context of the HTP reform implementation.

## Conclusions

The analysis presented in this paper shows that the Family Medicine Model in Turkey improved patient satisfaction across a range of satisfaction indicators. This study provides the first causally strong evidence that a major health systems transformation–the introduction of a Family Medicine Model–can increase patient satisfaction. Policy makers seeking to improve patient satisfaction through health system transformation should consider primary care approaches with elements similar to those used in Turkey’s transformation initiative.

## Supporting information

S1 Appendix Methodological notes on principal components analysis. (DOCX)

S2 Appendix Sensitivity analyses. (DOCX)

S1 Data Dataset to replicate econometric analysis. (DTA)

## Abbreviations

EUROPEP: European Patients Evaluate General/Family Practice
PC1: Principal Component 1
PC2: Principal Component 2
PHC: Primary Health Care
TPSHSS: Turkey Patient Satisfaction with Health Services Survey
