# Supplementary material for: The impact of the Family Medicine Model on patient satisfaction in Turkey: Panel analysis with province fixed effects
Source: PLoS One. 2019 Jan 30;14(1):e0210563. doi: 10.1371/journal.pone.0210563 (PMC6353549; doi:10.1371/journal.pone.0210563)
Supplement: S1 Appendix — (DOCX) [file pone.0210563.s001.docx]

**S1 Appendix. Methodological note on principal component analysis**

By using principal component analysis, we treat our data as continuous which imposes the assumption that the distances between the ordered categories are equal. As found by Kolenikov and Angeles (2009), this assumption is valid for our data because the response categories are ordered with respect to satisfaction levels and all input variables into the principal component analysis use the same scale [1-3]. We find the data across the 23 survey questions are internally consistent, with a Cronbach’s alpha of 0·97.

To identify the relevant principal components for additional analysis, we use a cut off of 1.0 to the component eigenvalues [4]. The eigenvalue calculated for a principal component represents the amount of variance in the total data accounted for by a factor. Only the first two components had an eigenvalue greater than 1.0 and combined they explain 65.13% of the total variance in the data (principal component 1 explains 48.75% and principal component 2 explains 16.38%). We use an orthogonal (varimax) rotation for the principal component results so that each variable loads as highly as possible on only one of the two components (see Table a for principal component factor loadings).

**Table a: Principal Component Factor Loading Results**

| Question | Outcome | PC1:  Clinical Behaviour | PC2:  Organization of Care |
| --- | --- | --- | --- |
| Q1 | Making you feel you had time during consultations | **0.27** | -0.05 |
| Q2 | Interest in your personal situation | **0.27** | -0.04 |
| Q3 | Making it easy for you to tell him or her about your problems | **0.28** | -0.07 |
| Q4 | Involving you in decisions about medical care | **0.26** | -0.04 |
| Q5 | Listening to you | **0.29** | -0.11 |
| Q6 | Keeping your records and data confidential | **0.24** | -0.05 |
| Q7 | Quick relief of your symptoms | **0.25** | -0.01 |
| Q8 | Helping you to feel well so that you can perform your normal daily activities | **0.25** | -0.01 |
| Q9 | Thoroughness | **0.28** | -0.07 |
| Q10 | Physical examination | **0.27** | -0.06 |
| Q11 | Offering you services for preventing diseases | **0.20** | 0.06 |
| Q12 | Explaining the purpose of test and treatments | **0.21** | 0.06 |
| Q13 | Telling you what you wanted to know about your complaints or disease | **0.23** | 0.03 |
| Q14 | Help in dealing with emotional problems related to your health status | **0.18** | 0.12 |
| Q15 | Helping you to understand the importance of following his or her advice | **0.21** | 0.09 |
| Q16 | Knowing what s/he had done or told you to do during previous contacts | **0.17** | 0.14 |
| Q17 | Preparing you for what to expect from referral to a specialists or hospital care | **0.16** | **0.16** |
| Q18 | The helpfulness of the staff (other than the doctor) | 0.09 | **0.23** |
| Q19 | Getting an appointment to suit you | 0.04 | **0.36** |
| Q20 | Getting through to the Family Health Centre on the phone | -0.05 | **0.51** |
| Q21 | Being able to speak to the GP on the telephone | -0.05 | **0.50** |
| Q22 | Waiting time in the waiting room | 0.03 | **0.35** |
| Q23 | Providing quick services for urgent health problems | 0.09 | **0.27** |

Factor loadings >0.15 are shown in bold font. The principal component “Clinical Behaviour” explains 49% of total variance, and the principal component “Organization of Care” explains 16% of total variance.

A survey question had to have a factor loading of 15% or greater to be considered relevant to a principal component. Factor loadings are the correlation between the original variables and the component factors estimated in the principal component analysis. A factor loading of 15% signifies that the principal component explains 15% of the variance in the original variable. Survey questions 1 through 17 having factor loadings greater than 15% for component 1, and survey questions 17 through 23 had factor loadings greater than 15% for component 2 (see Table a).

Our use of principal component analysis with ordered-categories imposes linearity assumptions that might introduce bias in our estimates. However, similar to the ordered-logit approach we assume that the distance between each category is equal. Therefore, our principal component results should be viewed as a linear approximation.

**Table b: Summary Statistics for Principal Components 1 and 2**

|  | 2010 | | 2011 | | 2012 | |
| --- | --- | --- | --- | --- | --- | --- |
|  | Mean | Standard Deviation | Mean | Standard Deviation | Mean | Standard Deviation |
| Principal Component 1 | -1.00 | 4.04 | 0.06 | 3.06 | 0.64 | 2.85 |
| Principal Component 2 | -0.52 | 2.34 | -0.09 | 1.82 | 0.43 | 1.66 |

**References**

1. Kolenikov S, Angeles G. Socioeconomic Status Measurement with Discrete Proxy Variables: Is Principal Component Analysis a Reliable Answer? Review of Income and Wealth. 2009;55(1):128-65.

2. Howe LD, Hargreaves JR, Huttly SR. Issues in the construction of wealth indices for the measurement of socio-economic position in low-income countries. Emerging Themese in Epidemiology. 2008;5(3).

3. Sharker MY, Nasser M, Abedin J, Arnold BF, Luby SP. The risk of misclassifying subjects within principal component based asset index. Emerging Themes in Epidemiology. 2014;11(6).

4. Jeffers JNR. Two Case Studies in the Application of Principal Component Analysis. Journal of the Royal Statistical Society Series C (Applied Statistics). 1967;16(3):225-36. doi: 10.2307/2985919.
