# Supplementary material for: The impact of the Family Medicine Model on patient satisfaction in Turkey: Panel analysis with province fixed effects
Source: PLoS One. 2019 Jan 30;14(1):e0210563. doi: 10.1371/journal.pone.0210563 (PMC6353549; doi:10.1371/journal.pone.0210563)
Supplement: S2 Appendix — (DOCX) [file pone.0210563.s002.docx]

**S2 Appendix. Sensitivity Analyses**

We conduct three additional sensitivity analyses to check the robustness of our results. First, we reran the estimations with the individual patient satisfaction variables as outcomes, using the largest possible sample without missing data for each particular patient satisfaction outcome (see Table c for results). Second, we imputed all missing values using ‘chained multiple imputations’, and then reran all estimations with the individual patient satisfaction variables as outcomes (see Table d for results) [1]. Third, we reassigned the eight provinces that had partially introduced family medicine-centred PHC between January 2010 and May 2010 to the set of control provinces rather than to the set of intervention provinces. We found the effect sizes shown in Fig 2 and Table c remained essentially unchanged (all coefficients changed by no more than 5%) and none of the determination of significance changed. These sensitivity analyses confirm the results presented in the paper. In the case of the imputed data, we find that all coefficients are highly significant due to the increased sample size.

**Table c: Regression Results: Satisfaction Questions, Complete Case Analysis of Data available for Each Estimation**

| # | Outcome | Adjusted Odds Ratio* | 95% Confidence Interval | p-value** | Observations |
| --- | --- | --- | --- | --- | --- |
| 1 | Making you feel you had time during consultations | 1.61 | 1.18 – 2.21 | 0.003 | 101,613 |
| 2 | Interest in your personal situation | 1.59 | 1.15 – 2.18 | 0.005 | 100,710 |
| 3 | Making it easy for you to tell him or her about your problems | 1.43 | 1.05 – 1.96 | 0.024 | 101,348 |
| 4 | Involving you in decisions about medical care | 1.49 | 1.07 – 1.06 | 0.17 | 100,535 |
| 5 | Listening to you | 1.35 | 0.98 – 1.85 | 0.065 | 101,540 |
| 6 | Keeping your records and data confidential | 1.28 | 0.93 – 1.77 | 0.129 | 96,394 |
| 7 | Quick relief of your symptoms | 1.35 | 1.00 – 1.81 | 0.045 | 100,926 |
| 8 | Helping you to feel well so that you can perform your normal daily activities | 1.42 | 1.05 – 1.92 | 0.024 | 100,287 |
| 9 | Thoroughness | 1.22 | 0.89 – 1.66 | 0.213 | 101,511 |
| 10 | Physical examination | 1.25 | 0.91 – 1.70 | 0.165 | 101,243 |
| 11 | Offering you services for preventing diseases | 1.25 | 0.92 – 1.71 | 0.150 | 98,120 |
| 12 | Explaining the purpose of test and treatments | 1.46 | 1.09 – 1.96 | 0.011 | 99,647 |
| 13 | Telling you what you wanted to know about your complaints or disease | 1.46 | 1.07 – 1.98 | 0.016 | 101,083 |
| 14 | Help in dealing with emotional problems related to your health status | 1.47 | 1.09 – 1.98 | 0.012 | 97,758 |
| 15 | Helping you to understand the importance of following his or her advice | 1.40 | 1.03 – 1.91 | 0.032 | 100,633 |
| 16 | Knowing what s/he had done or told you to do during previous contacts | 1.63 | 1.21 – 2.19 | 0.001 | 99,701 |
| 17 | Preparing you for what to expect from referral to a specialists or hospital care | 1.26 | 0.92 – 1.72 | 0.145 | 95,458 |
| 18 | The helpfulness of the staff (other than the doctor) | 1.10 | 0.82 – 1.47 | 0.530 | 100,728 |
| 19 | Getting an appointment to suit you | 1.81 | 1.25 – 2.62 | 0.002 | 87,292 |
| 20 | Getting through to the Family Health Centre on the phone | 1.90 | 1.28 – 2.82 | 0.001 | 81,228 |
| 21 | Being able to speak to the GP on the telephone | 2.31 | 1.60 – 3.35 | <0·0001 | 79,919 |
| 22 | Waiting time in the waiting room | 1.50 | 1.09 – 2.07 | 0.013 | 100,647 |
| 23 | Providing quick services for urgent health problems | 1.13 | 0.82 – 1.57 | 0.448 | 95,660 |

*The fixed effect estimations control for time, education, sex, age, and place of residence. Standard errors are clustered at the province level. This model imposes the proportional odds assumption, which means that we assume that the distance between each of the five Likert scale categories is equivalent. The number of observations varies across estimations because we use the sample of conduct a complete case analysis by question.

**By carrying out 23 estimations, we increase the probability of falsely identifying a significant result. To control for this Type I error, we can use the Bonferroni adjustment for our p-values. By doing so, we would only judge results to be significant if the p-value were less than 0·002.

**Table d: Regression Results: Satisfaction Questions, Using Imputed Data for Missing Observations**

| # | Outcome | Adjusted Odds Ratio* | 95% Confidence Interval | p-value** |
| --- | --- | --- | --- | --- |
| 1 | Making you feel you had time during consultations | 1.61 | 1.18 – 2.21 | 0.003 |
| 2 | Interest in your personal situation | 1.59 | 1.16 – 2.16 | 0.004 |
| 3 | Making it easy for you to tell him or her about your problems | 1.44 | 1.05 – 1.96 | 0.023 |
| 4 | Involving you in decisions about medical care | 1.49 | 1.08 – 2.05 | 0.015 |
| 5 | Listening to you | 1.35 | 0.98 – 1.85 | 0.064 |
| 6 | Keeping your records and data confidential | 1.28 | 0.95 – 1.72 | 0.105 |
| 7 | Quick relief of your symptoms | 1.35 | 1.01 – 1.80 | 0.043 |
| 8 | Helping you to feel well so that you can perform your normal daily activities | 1.42 | 1.05 – 1.90 | 0.022 |
| 9 | Thoroughness | 1.22 | 0.89 – 1.65 | 0.213 |
| 10 | Physical examination | 1.24 | 0.91 – 1.70 | 0.163 |
| 11 | Offering you services for preventing diseases | 1.26 | 0.93 – 1.68 | 0.137 |
| 12 | Explaining the purpose of test and treatments | 1.47 | 1.10 – 1.95 | 0.008 |
| 13 | Telling you what you wanted to know about your complaints or disease | 1.46 | 1.08 – 1.97 | 0.015 |
| 14 | Help in dealing with emotional problems related to your health status | 1.47 | 1.40 – 1.55 | <0·0001 |
| 15 | Helping you to understand the importance of following his or her advice | 1.40 | 1.03 – 1.89 | 0.029 |
| 16 | Knowing what s/he had done or told you to do during previous contacts | 1.63 | 1.22 – 2.17 | 0.001 |
| 17 | Preparing you for what to expect from referral to a specialists or hospital care | 1.26 | 0.95 – 1.68 | 0.104 |
| 18 | The helpfulness of the staff (other than the doctor) | 1.09 | 0.83 – 1.47 | 0.513 |
| 19 | Getting an appointment to suit you | 1.81 | 1.37 – 2.41 | <0·0001 |
| 20 | Getting through to the Family Health Centre on the phone | 1.91 | 1.44 – 2.55 | <0·0001 |
| 21 | Being able to speak to the GP on the telephone | 2.33 | 1.77 – 3.01 | <0·0001 |
| 22 | Waiting time in the waiting room | 1.50 | 1.09 – 2.06 | 0.011 |
| 23 | Providing quick services for urgent health problems | 1.13 | 0.84 – 1.53 | 0.405 |

*The fixed effect estimations control for time, education, sex, age, and place of residence. We use multiple imputations to calculate the missing observations by survey question using chained multiple imputations with ordered-logit models. There are 101,903 observations for each estimation, which is the total sample size for the EUROPEP-TPSHSS 2010, 2011, and 2012 combined. Standard errors are clustered at the province level. **By carrying out 23 estimations, we increase the probability of falsely identifying a significant result. To control for this Type I error, we can use the Bonferroni adjustment for our p-values. By doing so, we would only judge results to be significant if the p-value were less than 0·002.

**References**

1. Royston P, White IR. Multiple Imputation by Chained Equations (MICE): Implementation in Stata. The Journal of Statistical Software. 2011;45(4).
